# Type I Interferons Enhance the Repair of Ultraviolet Radiation-Induced DNA Damage and Regulate Cutaneous Immune Suppression

**DOI:** 10.3390/ijms23031822

**Published:** 2022-02-05

**Authors:** Mohammad Asif Sherwani, Israr Ahmad, Monica J. Lewis, Ahmed Abdelgawad, Harunur Rashid, Kevin Yang, Ching-Yi Chen, Chander Raman, Craig A. Elmets, Nabiha Yusuf

**Affiliations:** 1 Department of Dermatology, University of Alabama at Birmingham, Birmingham, AL 35294, USA; sherwani@uab.edu (M.A.S.); israrahmad@uabmc.edu (I.A.); mlewis1@uab.edu (M.J.L.); gawad@uab.edu (A.A.); hrashid@uab.edu (H.R.); kyang22@uab.edu (K.Y.); celmets@uabmc.edu (C.A.E.); 2 Department of Biochemistry and Molecular Genetics, University of Alabama at Birmingham, Birmingham, AL 35294, USA; cchen@uab.edu; 3 Division of Clinical Immunology and Rheumatology, University of Alabama at Birmingham, Birmingham, AL 35294, USA; chanderraman@uabmc.edu; 4 O’Neal Comprehensive Cancer Center, University of Alabama at Birmingham, Birmingham, AL 35294, USA; 5 Veteran Affairs Medical Center, Birmingham, AL 35294, USA

**Keywords:** ultraviolet radiation, type I interferons, skin, DNA repair, immune suppression

## Abstract

Type I interferons (IFNs) are important enhancers of immune responses which are downregulated in human cancers, including skin cancer. Solar ultraviolet (UV) B radiation is a proven environmental carcinogen, and its exposure contributes to the high prevalence of skin cancer. The carcinogenic effects of UV light can be attributed to the formation of cyclobutane pyrimidine dimers (CPD) and errors in the repair and replication of DNA. Treatment with a single dose of UVB (100 mJ/cm^2^) upregulated IFNα and IFNβ in the skin of C57BL/6 mice. IFNα and IFNβ were predominantly produced by CD11b+ cells. In mice lacking the type I IFN receptor 1 (IFNAR1), the repair of CPD following cutaneous exposure to a single dose of UVB (100 mJ/cm^2^) was decreased. UVB induced the expression of the DNA repair gene xeroderma pigmentosum A (*XPA*) in wild-type (WT) mice. In contrast, such treatment in IFNAR1 (*IFNAR1-/-*) mice downregulated *XPA*. A local UVB regimen consisting of UVB radiation (150 mJ/cm^2^) for 4 days followed by sensitization with hapten 2,4, dinitrofluorobenzene (DNFB) resulted in significant suppression of immune responses in both WT and *IFNAR1-/-* mice. However, there were significantly higher CD4+CD25+Foxp3+ regulatory T-cells in the draining lymph nodes of *IFNAR1-/-* mice in comparison to WT mice. Overall, our studies reveal a previously unknown action of type I IFNs in the repair of photodamage and the prevention of UVB-induced immune suppression.

## 1. Introduction

Ultraviolet (UV) B radiation (290–320 nm) is an important trigger for the suppression of immune responses and the initiation of non-melanoma skin cancers [1,2,3]. The molecular basis for these biological activities stems at least in part from its ability to damage DNA, predominantly in the form of cyclobutane pyrimidine dimers (CPD). When UVB-induced DNA damage occurs in cells, there is a meticulous attempt to repair it through the activation of DNA repair enzymes [4]. Xeroderma pigmentosum (XP), an autosomal recessive disorder of DNA repair, is associated with a greater than 1000-fold increase in the susceptibility to UVB-induced skin malignancies. The xeroderma pigmentosum complementation group A (XPA) is the most common type of XP and is caused by deficient nucleotide excision repair (NER) [4]. UVB-induced DNA damage is not only of importance in skin cancer but also plays a critical role in UVB-induced immune suppression [5]. UVB-induced DNA damage promotes the migration of antigen-presenting cells from the skin to the draining lymph node and impairs the capacity of antigen-presenting cells to present the antigen. Both of these activities contribute to an inability to initiate cell-mediated immune responses such as contact hypersensitivity (CHS) [6]. Sensitization with haptens through UVB-exposed skin does not result in CHS but induces hapten-specific tolerance. This tolerance is mediated through regulatory T-cells (Treg) [7]. There is accumulating evidence that UV-induced regulatory T cells belong to the CD4+CD25+ T cell subset [8,9,10].

Interferons (IFN) are cytokines that play a role in the regulation of proliferation, differentiation, and immune function [11]. IFNs play an important role in both innate and adaptive immunity and consist of three families (I, II, and III) of cytokines that bind to distinct cell-surface receptors [12]. The genes encoding type I IFN (primarily α and β) and type III (λ) IFN are induced in response to viral or bacterial infection [13]. The interferon regulatory factors (IRF) were first characterized as regulators of type I IFN genes. IFN-stimulated genes (ISG) are now known to have diverse roles in immunity [14,15,16,17]. IFN bind to cell-surface receptors that activate janus kinases (JAK) and the tyrosine phosphorylation of the signal transducer and activator of transcription (STAT) 1 and STAT2 [18,19]. All characterized type I IFNs transmit their signals through the type I IFN receptor composed of IFNAR1 and IFNAR2 components. IFNAR1 and IFNAR2 exist as heterodimers, and the loss of either subunit results in a complete abrogation of receptor function [20]. In addition to these indirect effects, type I IFNs directly regulate T-cell survival, proliferation, and differentiation into effector and memory cells. They also regulate B-cells [21,22].

A recent study delineated the role of type I IFNs in the regulation of UVB-induced cutaneous inflammatory responses. They reported a reduced inflammatory infiltrate and a downregulation of inflammatory genes when *IFNAR1*-deficient mice were exposed to UVB radiation [23]. A single exposure to UVB light triggers an early cutaneous IFN-1 response exaggerated in mice and humans. UVB light stimulated not only a local but also a systemic IFN-1 response in the blood and kidneys [24,25].

The TLR7 agonist imiquimod has been reported to promote the repair of UVB-induced CPDs and prevent UVB-induced immunosuppression in mice [26,27]. One of the mechanisms through which it mediates its effect is by the induction of type I IFNs. In this study, we evaluated the role of type I IFNs in the regulation of UVB-induced DNA damage and UVB-induced suppression of immune responses, and the mechanisms involved in this process.

## 2. Results

### 2.1. UVB Upregulates Type I IFNs and Transcription Factors IRF1 and IRF3

**Figure 1:**
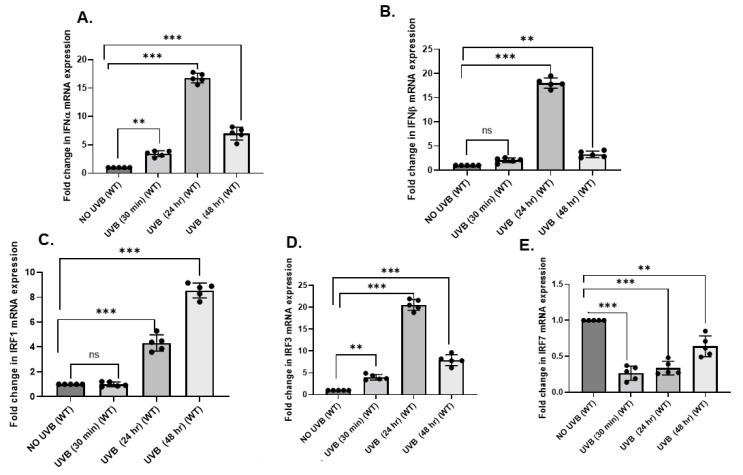
UVB-induces type I IFNs and IRFs. Panels of C57BL/6 mice were exposed to a single dose of UVB radiation (100 mJ/cm^2^), and thereafter sacrificed at 30 m, 24 h, and 48 h and analyzed for the mRNA expression for *IFNα*, *IFNβ*, *IRF1*, *IRF3*, and *IRF7* in the skin using a quantitative real-time PCR (qPCR) with custom primers. There was an increase in *IFNα*, *IFNβ*, *IRF1*, and *IRF3* mRNA expression with increasing time post-UV exposure, as determined by a quantitative real-time PCR (qPCR) in the skin of mice than in the UV-unexposed skin of mice (**A**–**E**). Experiments were conducted and repeated separately in 5 animals in each group with identical results. (** *p* < 0.01, and *** *p* < 0.001 and ns, not significant).

To investigate the induction of type I interferons (IFNα/β) and/or interferon regulatory factors (IRFs), the dorsal skin of C57BL/6 mice was exposed to UVB radiation (100 mJ/cm^2^). Mice were then sacrificed at various time intervals (30 m, 24 h, and 48 h) and their skin was analyzed for *IFNα* and *IFNβ*, *IRF1*, *IRF3*, and *IRF7* using a quantitative real-time PCR (qPCR). The expression of *IFNα* and *IFNβ* was significantly upregulated (*p* < 0.001) after 24 h and 48 h post-UVB exposure (Figure 1A,B). The expression of *IRF1* and *IRF3* also significantly increased (*p* < 0.001) (Figure 1C,D), while *IRF7* was suppressed by post-UVB exposure (Figure 1E).

### 2.2. CD11b+ Cells Are the Major Producers of UVB-Induced Type I IFNs in the Skin

**Figure 2:**
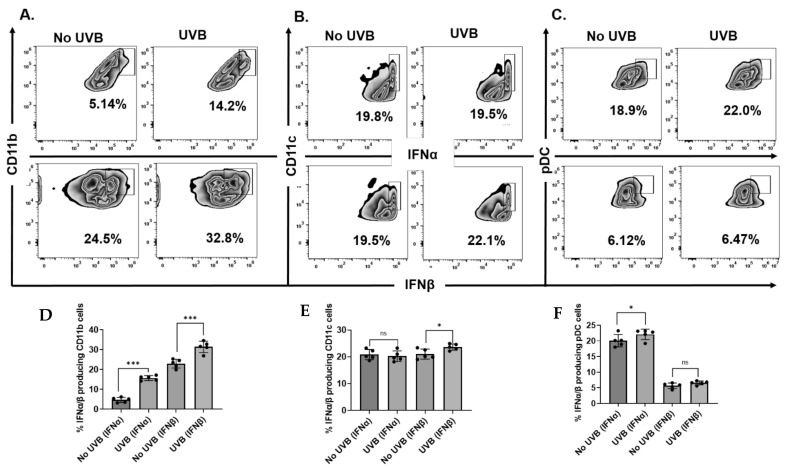
Exposure to UVB causes the production of type I IFNs mainly by CD11b cells. Panels of C57BL/6 mice were sacrificed 24 h post-UVB (100 mJ/cm^2^) exposure and single-cell suspensions of skin (dermal/epidermal) were prepared from dorsal skin. Cells were stained with anti-CD11b (**A,D**), anti-CD11c (**B,E**), anti-pDC (**C,F**), anti-MHCII, and anti-IFNα/β followed by AF488 conjugated secondary antibodies and analyzed using flow cytometry. Experiments were conducted and repeated separately in 5 animals in each group with identical results. (* *p* < 0.05, *** *p* < 0.001 and ns, not significant).

To identify the type I IFN-producing cells in mice after UVB radiation, the dorsal shaved skin of C57BL/6 mice (N = 5 per group) was exposed to a single dose of UVB radiation (100 mJ/cm^2^). Skin was harvested from mice 24 h after UVB exposure, single-cell suspensions were prepared by enzymatic digestion as described previously [22], and stained with anti-CD11b, anti-CD11c, anti-PDCA-1, and anti-MHC II antibodies followed by intracellular staining with anti-IFNα and anti-IFNβ antibodies. Among the innate immune cells, CD11b^+^ cells exhibited the greatest UVB-induced expression of I IFNs (Figure 2A). Although CD11c+ dendritic cells (DC) and plasmacytoid dendritic cells (pDC) expressed high levels of IFNα and IFNβ, their expression was not altered by UVB (Figure 2B,C).

### 2.3. UVB-Induced DNA Damage Persisted in IFNAR1-Knockout Mice

**Figure 3:**
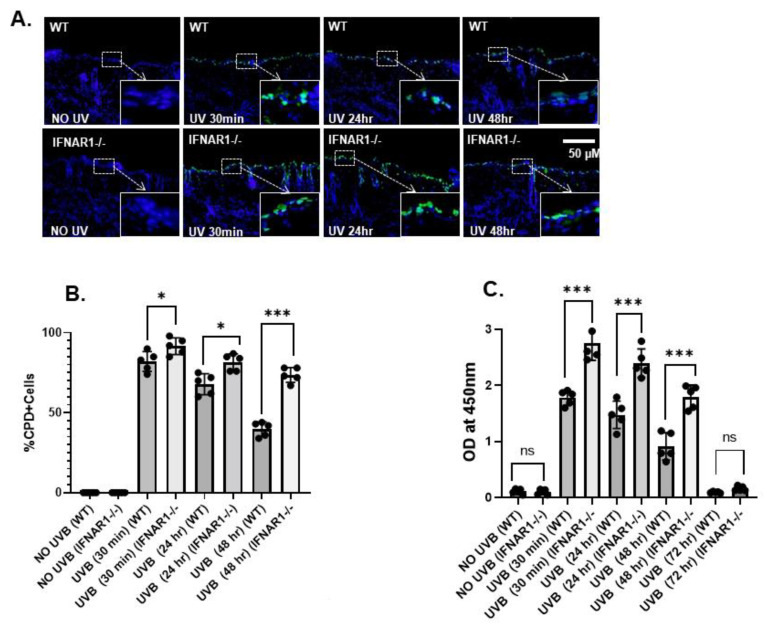
IFNAR1 deficiency prevents the repair of UVB-induced CPD. (**A**). Panels of *IFNAR1-/-* and wild-type (WT) mice were exposed to a single dose of UVB radiation (100 mJ/cm^2^) and thereafter sacrificed at 30 min, 24 h, and 48 h. Frozen skin sections (5 μm thick) were subjected to immunostaining to detect CPD+ cells that were stained green. CPD was not detected in non-UV-exposed skin. Magnification, 40×. (**B**). Summary of CPD+ cells. The number of CPD+ cells was counted in five to six different areas of the sections under an Olympus BX41 microscope, and the numbers reported represent the percentage of CPD+ cells ± SD in the epidermis. (**C**). Quantification of CPD using ELISA. Experiments were conducted and repeated separately in 5 animals in each group with identical results. Scale bar = 50 μM. (* *p* < 0.05, and *** *p* < 0.001 and ns, not significant).

To determine the effect of type I IFNs on UVB-induced DNA damage, *IFNAR1-/-* and WT mice were exposed to UVB radiation (100 mJ/cm^2^) on their dorsal skin. Skin was harvested from mice and cells were stained for CPD. CPD+ cells were increased in *IFNAR1-/-* mice at each time point examined, and the difference was significant at 24 and 48 h after exposure to UVB (Figure 3A,B). The presence of a significantly (*p* < 0.001) greater number of CPDs in the skin of UVB-treated *IFNAR1-/-* mice in comparison to WT mice was independently confirmed by ELISA (Figure 3C).

### 2.4. Efficiency of DNA Repair Is Decreased in IFNAR1 Knockout Mice

**Figure 4:**
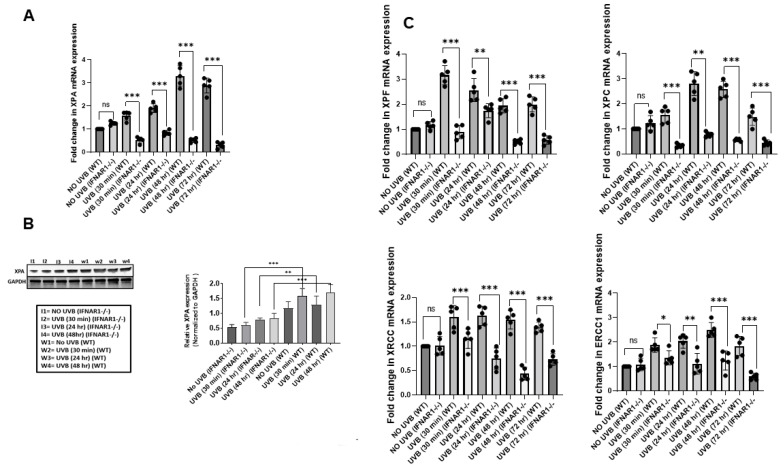
*IFNAR1* knockout mice have a low expression of XPA. *IFNAR1-/-* and WT mice were sacrificed at 30 m, 24 h, 48 h, and 72 h after UVB exposure. Skin samples were collected and RNA was isolated and subjected to *XPA* mRNA expression analysis using a real-time PCR. (**A**). There was an increase in *XPA* mRNA expression with increasing time post-UV exposure, as determined by a quantitative real-time PCR (qPCR) in the skin of WT mice than in the UV-exposed skin of *IFNAR1-/-* mice. (**B**). There was increased XPA protein expression with an increase in time post-UV exposure as determined by western blot analysis, and this increase was more prominent in the skin of WT mice than in the UV-exposed skin of *IFNAR1-/-* mice. Lane 1 represents the group of animals not exposed to UVB whereas lanes 2, 3, and 4 represent time points 30 min, 24 hr, and 48 hr post-UVB exposure, respectively. (**C**). There was an increase in *XPF*, *XPC*, *XRCC*, and *ERCC1* mRNA expression with increasing time post-UV exposure, as determined by a quantitative real-time PCR (qPCR) in the skin of WT mice than in the UV-exposed skin of *IFNAR1-/-* mice. Experiments were conducted and repeated separately in 5 animals in each group with identical results. (* *p* < 0.05, ** *p* < 0.01, and *** *p* < 0.001 and ns, not significant).

We next investigated whether the decreased DNA repair in the absence of IFNAR1 was due to the attenuated stimulation of nucleotide excision repair (NER) that can be assayed by measurement of *XPA* expression [4]. *IFNAR1-/-* and WT mice were exposed to UVB and sacrificed at 30 m, 24 h, 48 h, and 72 h after exposure. Skin samples were collected and the expression of *XPA* was determined by a qPCR. UVB exposure significantly reduced (*p* < 0.001) the expression of *XPA* mRNA and protein in the skin of *IFNAR1-/-* mice in comparison to WT mice (Figure 4A,B). Similar results were obtained for *XPC, XPF, ERCC1, and XRCC1* mRNA expression by qPCR (Figure 4C).

### 2.5. IFNAR Signaling Is Not Necessary for UVB-Induced Immune Suppression

**Figure 5:**
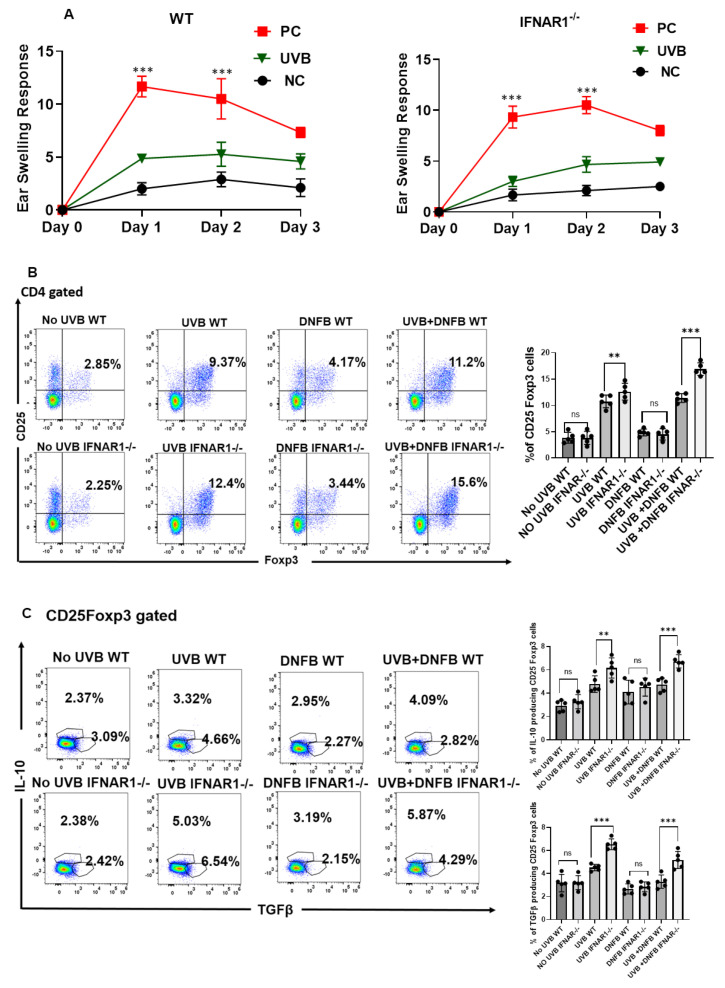

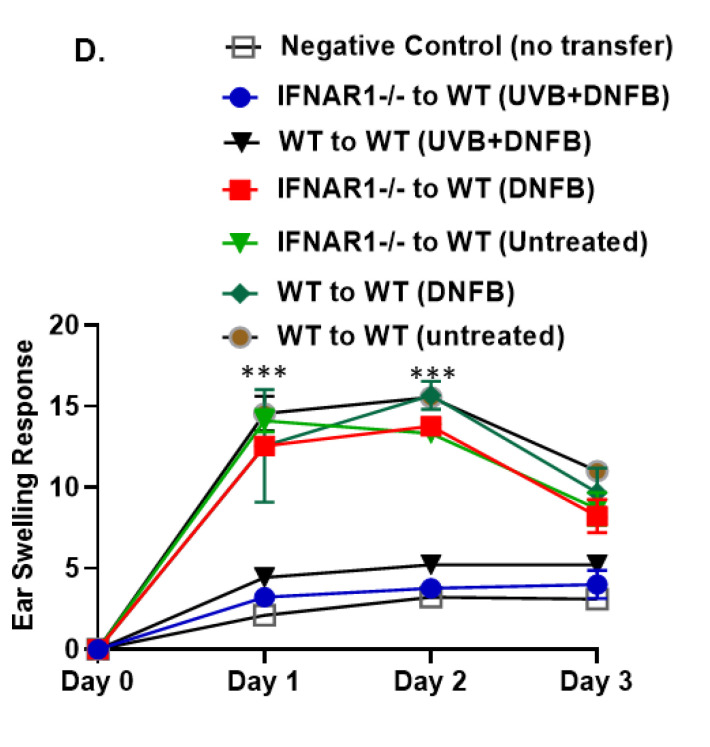
IFNAR1 deficiency does not have a significant effect on UVB-induced immune suppression. Panels of *IFNAR1-/-* and wild-type (WT) mice were exposed to UVB radiation (100 mJ/cm^2^) followed by treatment with DNFB as described in the Methods section. Positive control mice (PC) were not exposed to UVB but sensitized with DNFB whereas negative control mice (NC) were neither exposed to UVB nor sensitized with DNFB. (**A**). The development of DNFB contact hypersensitivity was significantly suppressed (*p* < 0.001) by UVB exposure in both *IFNAR1-/-* and WT mice. (**B**). CD4+CD25+Foxp3+ regulatory T-cells were stained for flow cytometry from the draining lymph nodes of *IFNAR1-/-* and WT mice. The number of CD4+CD25+Foxp3+ regulatory T-cells was significantly higher in *IFNAR1-/-* mice in comparison to WT mice. (**C**). The CD4+CD25+Foxp3+ regulatory T-cells secreted IL-10 and TGFβ stained for flow cytometry from the draining lymph nodes of *IFNAR1-/-* and WT mice. The CD4+CD25+Foxp3+ gated regulatory T-cells from *IFNAR1-/-* mice secreted more IL-10 and TGFβ in comparison to cells from WT mice. (**D**). WT and *IFNAR1-/-* mice mice were irradiated with UVB (100 mJ cm^2^) on shaved back skin once a day for four consecutive days. The mice were sensitized once with DNFB on the UVB-treated skin area 24 h after the last UVB. Control mice that were not treated with UVB were also sensitized with DNFB. The spleen and the draining lymph node cells from all sensitized mice were harvested 5 days later. CD4+ T cells were purified and transferred i.v. into naïve wild-type mice (5 × 10^6^ cells/mouse), that were not treated with UVB. The recipient mice were sensitized with DNFB 24 h later and challenged 5 days after the sensitization There were 5 mice per group and the experiment was repeated twice. Results are expressed as mean± SD from both experiments. (** *p* < 0.01, and *** *p* < 0.001 and ns, not significant).

UVB exposure suppresses cell-mediated immune responses [5]; therefore, we investigated whether *IFNAR1* signaling was necessary for UVB-induced immunosuppression. We found that UVB significantly suppressed CHS responses equally in WT and *IFNAR1-/-* mice (*p* < 0.001) (Figure 5A). Remarkably, the number of CD4+CD25+Foxp3+ Treg cells was significantly higher in *IFNAR1-/-* mice in comparison to WT mice (Figure 5B). The Treg cells from *IFNAR1-/-* mice also secreted more IL-10 and TGFβ than Treg cells from WT mice (Figure 5C).

### 2.6. IFNAR1 Signaling Inhibits the Development of UVB-Induced CD4+ Regulatory T Cells

UVB-induced regulatory cells (CD4+CD25+Foxp3+) have important roles in UVB- induced immune suppression and skin carcinogenesis [28]. They are able to transfer antigen-specific immune suppression to normal mice who are not treated with UVB. As we found in our immune suppression study, there were higher numbers of CD4+CD25+Foxp3+ Treg cells in *IFNAR1-/-* mice in comparison to WT mice (Figure 5B). We further investigated whether IFNAR1 signaling inhibits the development of UVB- induced regulatory T cells. Our results show that the transfer of CD4+T cells from UVB-treated IFNAR1-/- mice inhibited the CHS response in the recipient wild-type mice who were not treated with UVB (Figure 5D).

### 2.7. Type I IFNs Prevent UVB-Induced Immune Suppression by the DNA Repair Mechanism

**Figure 6:**
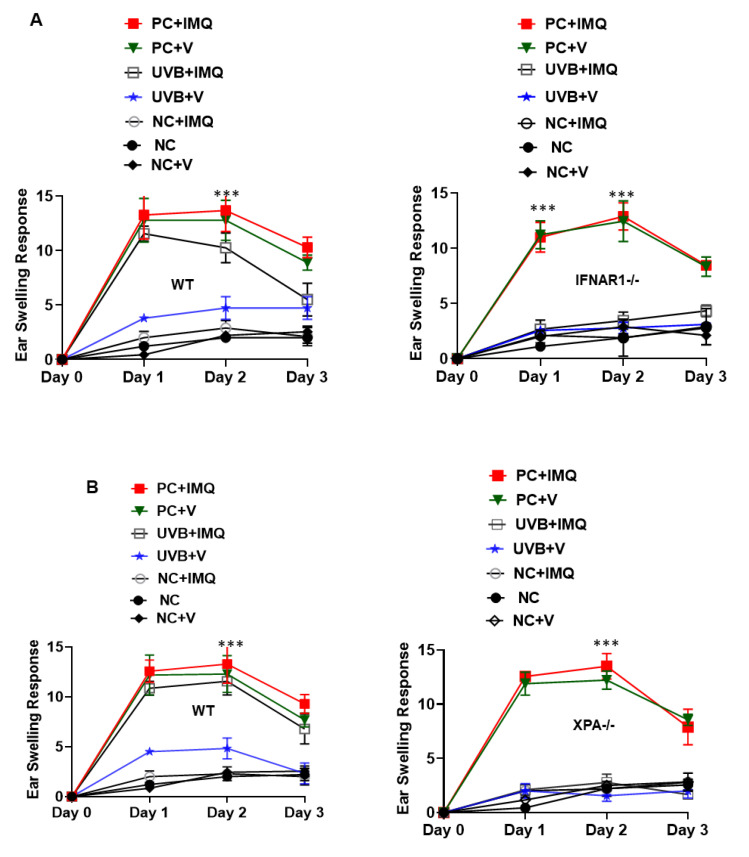
Augmentation of type I IFNs prevented UVB-induced immune suppression by DNA repair mechanism. (**A**) Panels of *IFNAR1-/-* and WT mice were treated with 5% imiquimod and were subjected to UVB-induced immunosuppression protocol as described in the Methods section. Positive control mice (PC) were not exposed to UVB but sensitized with DNFB whereas negative control mice (NC) were neither exposed to UVB nor sensitized with DNFB. Treatment with imiquimod was able to prevent UVB-induced immune suppression in WT mice but not in *IFNAR1-/-* mice (*** *p* < 0.001). (**B**) To determine whether this process was mediated via DNA repair, a similar experiment was performed in *XPA-/-* and WT mice. Imiquimod was able to inhibit UVB-induced immune suppression in WT mice (*** *p* < 0.001) but not in *XPA-/-* mice. Experiments were conducted and repeated separately in five animals in each group with identical results.

To assess whether the augmentation of type I IFNs mediated the prevention of UVB-induced immune suppression, WT and *IFNAR1-/-* mice were treated with 5% imiquimod or vehicle cream. The mice were subjected to UVB-induced immune suppression. Topical application of 5% imiquimod was able to inhibit immune suppression in WT mice (*p* < 0.001) but not in *IFNAR1-/-* mice (Figure 6A). Furthermore, to determine the role of DNA repair in this process, a panel of NER-deficient *XPA-/-* mice were treated with imiquimod and were subjected to UVB-induced immunosuppression protocol. Activation of type I IFNs by imiquimod was able to prevent UVB-induced immune suppression in WT mice but not in *XPA-/-* mice, who showed significant suppression of immune responses (*p* < 0.001) (Figure 6B).

Transfection of normal and UVB-treated plasmid in keratinocytes in the presence or absence of IMQ showed that the UVB-treated pEGFP plasmid gene could be repaired. The UVB-treated plasmid showed a 35% decreased expression documented by microscopy and FACS analysis compared to the control plasmid (Figure S1A,B). The decreased expression of UVB-treated pEGFP plasmid was fully rescued in the presence of IMQ. These results were further confirmed in a separate experiment where pEGFP was co-transfected with untreated DsRedexpress plasmid (Figure S1C,D). Again, we found around a 50% decreased expression of UVB-treated pEGFP which was rescued if the cells were treated with IMQ overnight prior to transfection. The untreated DsRedexpress plasmid showed comparable expression with all combination. Together, our data showed that UVB exposure causes damage in the gene, which can be repairable in the presence of IMQ.

## 3. Discussion

UVB-induced mutations in tumor suppressor genes are a major cause of the initiation of skin cancer. In this study, we showed that the signaling of type I IFNs (IFN-α/βsignaling has a necessary role in the repair of the DNA damage through induction of the DNA repair gene, *XPA*. Studies on pre-malignant actinic keratoses suggest that the expression of the suppressed interferon stimulated gene factor 3 (ISGF3) occurs early in human skin cancer development and that a reduced response to type I IFNs (IFN-α/β) is involved in the earliest stages of skin carcinogenesis [29,30].

A recent study delineated the role of type I IFNs in the regulation of UVB-induced cutaneous inflammatory responses. They reported a reduced inflammatory infiltrate and a downregulation of inflammatory genes when *IFNAR1*-deficient mice were exposed to UVB radiation [23]. There is no information on the role of type I IFNs in the repair of UVB-induced DNA damage. The IFN arm of the DNA damage response may have evolved as an antiviral mechanism in reaction to DNA damage induced by viruses, to reduce cellular proliferation and allow DNA repair, or as a mechanism to promote the death of cells with irreparable damage [31]. In this study, we presented a previously unknown role of type I IFNs in the regulation of UVB-induced DNA damage. Our data showed that in the absence of IFNAR1, there was a defect in DNA repair in mice. There were significantly more CPDs in *IFNAR1*-knockout mice.

The IFN regulatory factors (IRFs) were first characterized as transcriptional regulators of type I IFN genes and IFN-stimulated genes (ISGs) and are now known to have diverse roles in immunity [14,15,16,17]. Our data indicate that various IRFs 1 and 3 were upregulated in mice exposed to UVB radiation.

Soon after the discovery of IFN as an antiviral agent and the identification of multiple types of IFN, it was determined that type I IFNs were produced by and acted upon immune cells. Plasmacytoid dendritic cells (pDC) have been characterized as major producers of rapid and high levels of type I IFNs in viral infection largely owing to their high constitutive levels of the transcription factor IRF7 [32]. Type I IFNs are also produced by and regulate the activity of innate immune cells including macrophages, natural killer cells as well as antigen-presenting cells such as pDC [33]. The consequences of these interactions are the indirect effects of IFNs on the development of adaptive responses, via innate immune cell chemokines and cytokines. A study by Yin et al. found that pDCs were the major source of type I IFNs in mouse skin when they were exposed to two high doses of UVB radiation [34]. In our study, we found that the CD11b+ cells, and not the pDCs were major producers of type I IFNs. Our findings are in accordance with another study in which inflammatory monocytes were reported to be the major type I IFN-producing cells when mice were exposed to subacute doses of UVB radiation [23].

Type I IFNs have been reported to prevent the suppression of immune responses in several disease models. In a model of murine lupus, type I IFNs increased T-cell activation by suppressing regulatory T-cells [35]. An IFN-α antagonist, along with cyclosporine was able to prolong allograft survival in cynomolgus monkeys [36]. Studies have shown that an FDA-approved high dose interferon regimen administered intravenously in patients with stage 3–4 melanoma reduced the number of regulator T-cells in the peripheral blood [37]. Several studies revealed that regulatory T cells critically contribute to UVB-mediated tolerance and are able to transfer antigen-specific immune suppression to normal animals who are not treated with UVB [2,3,28]. We investigated the role of IFNAR1 signaling in the development of regulatory CD4+ T cells. In accordance with the literature [2,3,28], our results show that the transfer of CD4+T cells from UVB-treated *IFNAR1-/-* mice showed more inhibition of the CHS responses in the recipient wild-type mice who were not treated with UVB. The TLR7 agonist was able to repair DNA damage and prevent immune suppression in mice [26,27]. We have shown here that the TLR7-mediated inhibition of UVB immune suppression is mediated by DNA repair, possibly mediated by type I IFNs. However, several other cytokines and chemokines have been reported to be induced by imiquimod along with IFNα [37]. IL-12 is one such cytokine that has shown efficacy in DNA repair and the prevention of UVB-induced immune suppression [8]. A TLR7 agonist conjugate was able to elicit CD8+ T-cell responses by coordinating the recruitment and activation of both tissue-derived and lymphoid organ-resident dendritic cell subsets through a type I IFN and IL-12 co-dependent mechanism [38]. The results with imiquimod were further confirmed in *IFNAR1-/-* mice. Our data confirm the role of type I IFNs in the prevention of UVB-induced immune suppression via the suppression of regulatory T-cells. We have also shown that type I IFNs are able to prevent immune suppression via a DNA repair mechanism.

There are reports on the role of type I IFNs in the treatment of melanoma but there is limited information on type I IFNs in the treatment of non-melanoma skin cancers such as squamous cell carcinoma (SCC) and basal cell carcinoma (BCC). There is a gap in knowledge on the cellular and molecular mechanisms mediated by type I IFNs in development of SCC, BCC, and melanoma [39]. Future studies from our laboratory will delineate the role of type I IFNs in development of melanoma and non-melanoma skin cancer.

The findings of this study give us insight into the role of type I IFNs in the repair of UVB-induced DNA damage and immune suppression. We have shown that type I IFNs could be possible targets to develop novel drugs for the repair of UVB-induced DNA damage and the prevention of non-melanoma skin cancer.

## 4. Materials and Methods

Animals and reagents. *IFNAR1-/-* mice and wild type (WT) C57BL/6 were purchased from Jackson Laboratories (Bar Harbor, ME, USA). Both male and female mice used for experiments were 6 to 8 weeks of age. All animal procedures were performed according to National Institute of Health (NIH) guidelines under protocols approved by the Institute Animal Care and Use Committee of the University of Alabama at Birmingham (Protocol # 20631, last approved on 21 September 2020). The hapten 1-fluoro-2, 4-dinitrobenzene (DNFB) was purchased from Sigma-Aldrich (St. Louis, MO, USA). Aldara (5% imiquimod) was purchased from 3M Pharmaceuticals (St. Paul, MN, USA).

UVB light source and irradiation of mice. The dorsal shaved skin of panels of wild type (WT) C57BL/6 or *IFNAR1-/-* mice (N = 5 per group) were exposed to a single dose of UVB radiation (100 mJ/cm^2^) for DNA damage experiments. The UV emission source (Daavlin, UVA/UVB Research Irradiation Unit, Bryan, OH, USA) was equipped with a bank of four UVB lamps and an electronic controller to regulate UVB dosage at the fixed distance of 24 cm from the lamps to the dorsal skin surface of the mice. Wavelengths <290 nm were filtered out using Kodacel cellulose film (Eastman Kodak Co., Rochester, NY, USA). The majority of the resulting wavelengths were in the UVB (290–320 nm; ~80%) and UVA (~20%) range, with peak emission at 314 nm as monitored regularly.

### 4.1. Detection of CPD+ Cells in Skin Sections

UVB-induced DNA damage in the form of CPD^+^ cells was detected using a protocol described previously with some modifications [38]. Briefly, frozen skin sections (5 μm thick) were thawed and kept in 70 mM NaOH in 70% ethanol for 2 min to denature nuclear DNA, followed by neutralization for 1 min in 100 mM Tris-HCl (pH 7.5) in 70% ethanol. The sections were washed with a PBS buffer and incubated with 10% goat serum (in PBS) prior to incubation with a monoclonal antibody specific for CPD (Kamiya Biomedical Company, Seattle, WA, USA) or its isotype control (IgG1). The bound anti-CPD antibody was detected by incubation with Alexa Flour 488 labeled secondary antibody and counterstained by DAPI. CPD^+^ cells were counted under Olympus BX41 microscope in 5–6 different fields.

### 4.2. CPD Quantitation by ELISA

*IFNAR1-/-* and WT mice were irradiated with a single dose of 100 mJ/cm^2^ and skin was harvested at 30 m, 24 h, and 48 h post-UVB irradiation. Mice that were not exposed to UVB were used as controls. The skin tissue was washed, and the DNA was extracted using a genomic DNA DNeasy Blood and Tissue kit (Qiagen). CPDs were quantified using the STA-322 DNA damage ELISA kit (Cell Biolabs) according to the manufacturer’s protocol.

### 4.3. Isolation of Cells from the Lymph Nodes and Spleen

The draining lymph nodes were harvested from panels of mice after exposure to UVB and single-cell suspensions were prepared after digestion according to the published protocol [40]. Cells were stained for CD4, CD25, Foxp3, IL-10, TGFβ and analyzed using a flow cytometer (Attune NxT, Thermofisher Scientific, Waltham MA, USA) as described [25], and the analysis was performed using FlowJo software version 10.6.1.

### 4.4. Antibodies

Monoclonal antibodies used for flow cytometry studies included anti-mouse CD16/CD32 (2.4G2; Purified), CD4 (RM4-5; AF700, PerCP-Cy5.5 and GK1.5; AF488), CD25 (PC61.5; APC, AF700), Foxp3 (FJK-16s; PE), IL-10 (JES5-16E3; PerCp), TGFβ (TW7-16B4; PECy7) CD11b (M1-70 PerCp), CD11c(N418; APC) and PDCA-1(129C1,PE), MHC II (M5 114.15.2; AF700), IFNα and anti-IFNβ. These were purchased from (Thermofisher Scientific, Waltham, MA, USA).

### 4.5. UVB-Induced Suppression of the Contact Hypersensitivity (CHS) Response

Panels of *IFNAR1-/-* and WT mice were exposed to UVB (150 mJ/cm^2^) for four days. Twenty-four hours after the last exposure, 50 ul of 0.5% DNFB in acetone/olive oil (4:1) was applied to the shaved dorsal skin of mice. Five days later, both ears of mice were challenged with 10 ul of 0.2% DNFB in acetone/olive oil (4:1) per ear. Baseline ear thickness was measured before the application of DNFB on the ears. The increase in ear thickness was calculated for 3 days at 24 h intervals using an engineer’s micrometer (Dial Thickness gauge, Mitutoyo, Japan). To quantify the magnitude of the CHS response, increases in ear thickness were measured by subtracting the baseline ear thickness measurements from the measurements at 24, 48, and 72 h. Mice that were sensitized and challenged with DNFB served as positive controls, whereas mice that were challenged only on their ears served as negative controls [41,42].

### 4.6. Flow Cytometry Analysis of Skin Cells

Dorsal skin was harvested from panels of mice after exposure to UVB and single-cell suspensions were prepared after digestion, according to published protocols [26]. Cells were stained with anti-CD11b, anti-CD11c, anti-PDCA1, anti-MHC II, and anti-IFNα/β antibodies with AF-488 conjugated secondary antibody. The percentage of cells that expressed high levels of IFNα and IFNβ were analyzed. All the cell populations were analyzed in a flow cytometer (Attune NxT, Thermofisher Scientific, Waltham, MA, USA) as described [28] and the analysis was performed using FlowJo software version 10.6.1.

### 4.7. Preparation of Cell/Tissue Lysates and Western Blot Analysis

For the Western blot analysis of different proteins in skin samples, lysates were prepared using 0.05 mL of RIPA buffer containing 20 mM HEPES, pH 7.4, 2 mM EDTA, 250 mM NaCl, 0.1% NP-40, 2 mg/mL leupeptin, 2 mg/mL aprotinin, 1 mM PMSF, 0.5 mg/mL benzamidine, 1 mM dithiothreitol, and 1 mM sodium vanadate. About 50–80 µg protein was loaded in each well and resolved on 10–12% SDS-polyacrylamide gel and transferred onto PVDF membranes. Membranes were incubated in a blocking buffer for 2 h and then incubated with the primary antibodies in a blocking buffer for 2 h at room temperature or overnight at 4 °C. The membrane was then washed with TBS-T and incubated with a secondary antibody conjugated with horseradish peroxidase. Protein bands were visualized using the enhanced chemiluminescence detection system (iBright Western Blot imaging systems, Thermofisher Scientific, Waltham, MA, USA). To verify the equal protein loading and the transfer of proteins from gel to membrane, the blots were stripped and reprobed for GAPDH [38]. The band density was analyzed using IMAGE J software provided by the NIH and the values were normalized to the GAPDH band density.

### 4.8. RNA Extraction and Quantitative Real-Time PCR (qPCR)

Total RNA from the skin samples was extracted using a Trizol reagent (Life Technologies, Carlsbad, CA, USA) according to the manufacturer’s protocol. cDNA was synthesized from 1 µg RNA using iScript cDNA synthesis kit (Bio-Rad, Hercules, CA, USA) according to the manufacturer’s instructions. Using iQ^TM^ SYBR Green Master Mix (Bio-Rad, Hercules, CA, USA), cDNA was amplified by a real-time PCR with a Bio-Rad MyiQ thermocycler and SYBR Green detection system (Bio-Rad, Hercules, CA, USA). The standard PCR conditions were 95 °C for 10 min and then 40 cycles at 95 °C for 30 s, 60 °C for 30 s, and 72 °C for 30 s. The expression of XPA, IFNα, IFNβ, IRF1, IRF4, IRF7 gene (Table 1) was normalized to the expression level of the GAPDH mRNA in each sample (23, 40, 44–50). For mRNA analysis, the calculations for determining the relative level of gene expression were made using the cycle threshold (*C*_t_) method. The mean *C*_t_ values from duplicate measurements were used to calculate the expression of the target gene with normalization to a housekeeping gene used as internal control and using the formulae 2^−^^ΔΔCT^.

### 4.9. Transfection of Keratinocyte with Irradiated Plasmid for Microscopy and Flow Cytometry Analysis

To assess the effect of type I IFN inducer imiquimod (IMQ) on UVB-treated plasmid DNA, DsRedexpress and pEGFP plasmid (Addgene, Watertown, MA, USA) were used to transfect human keratinocyte cell line (HaCaT). To assess the effect of UVB, pEGFP plasmid was exposed to 5 kJ/m2 UVB prior to transfection [50]. Keratinocytes were transfected using PolyJet transfection reagent as per the manufacturer’s instructions ((SignaGen Laboratories, Rockville, MD, USA). Keratinocytes were plated in a 12-well plate and transfected with 500 ng of UVB treated pEGFP expression. A parallel plate was treated with IMQ for overnight prior to transfection with pEGFP. For control, pEGFP plasmid without UVB treatment were used. For the second experiment, DsRedexpress plasmid without UVB treatment were co-transfected with pEGFP plasmid. Briefly, DNA-PolyJet complexes were made in serum-free Opti-MEM media by mixing the plasmid DNA with PolyJet at 1:3 ratio for 20 m at 22 °C. The lipid–DNA complexes were overlaid on cells for 24 h at 37 °C. The pictures were captured using a Keyence microscope. Cells were harvested in PBS with 3% FBS for flow cytometry analysis.

Statistical analysis. In all experiments, UVB exposed and unexposed groups were compared separately using two-way analysis of variance (ANOVA). All quantitative data are shown as the means ± SD. In each case, a value of *p* < 0.05 was considered to be statistically significant.

## Figures and Tables

**Table 1:** Primer sequences used in reverse transcription–polymerase chain reaction.

Gene	Primer Sequence	References
IFNα	5′-AGTGAGCTGACCCAGCAGAT-3′ 5′-AGTGAGCTGACCCAGCAGCAGAT-3′	[23]
IFNβ	5′-AAGAGTTACACTGCCTTTGCCATC-3′ 5′-CACTGTCTGCTGCTGGAGTTCATC-3′	[43]
IRF1	5′-CAGAGGAAAGAGAGAAAGTCC-3′ 5′-CACACGGTGACAGTCCTGG-3′	[44]
IRF3	5′-TGGGCAGCACAGCTGACATGA-3′ 5′-GCCCATTGCCCAGCCCTT-3′	[44]
IRF7	5′-TGGGGCCATGGGGCTGTA-3′5′-GCCTTGGGTTCCTGGATGTGA-3′	[44]
XPF/ERCC4	5’-GCAACAAGCCGAATACTCGT-3’ 5’-GTGTCAAAGGCAACAGCGT-3	[45]
XPC	5’-ACGTCCCAGGGAGAACGTAT-3’ 5’-TCCTCTGCGACCATCCCTTT-3’	[46]
XRCC	5′-CAGACAGCACACATCTCATC-3′ 5′-ACCCTCCTCAGTTCATCCT-3′	[47]
ERCC1	5’-GTGCAGAGGAAGCAGGGCGG-3’ 5-‘CAGGAGGGTCTGGCTGTCGGT-3’	[48]
GAPDH	5′-AACTTTGGCATTGTGGAAGG-3′ 5′-ACACATTGGGGGTAGGAACA-3′	[49]
XPA	5′-CAAAGGTGGCTTCATTTTAG-3′ 5′-GGTACATGTCATCTTCTAAG-3′	[49]

## Supplementary Materials

The following are available online at https://www.mdpi.com/article/10.3390/ijms23031822/s1.
