# Supplementary material for: Type I Interferons Enhance the Repair of Ultraviolet Radiation-Induced DNA Damage and Regulate Cutaneous Immune Suppression
Source: Int J Mol Sci. 2022 Feb 5;23(3):1822. doi: 10.3390/ijms23031822 (PMC8836948; doi:10.3390/ijms23031822)
Supplement: Supplementary file 1 [file ijms-23-01822-s001.zip › ijms-1574757-supplementary.pdf]

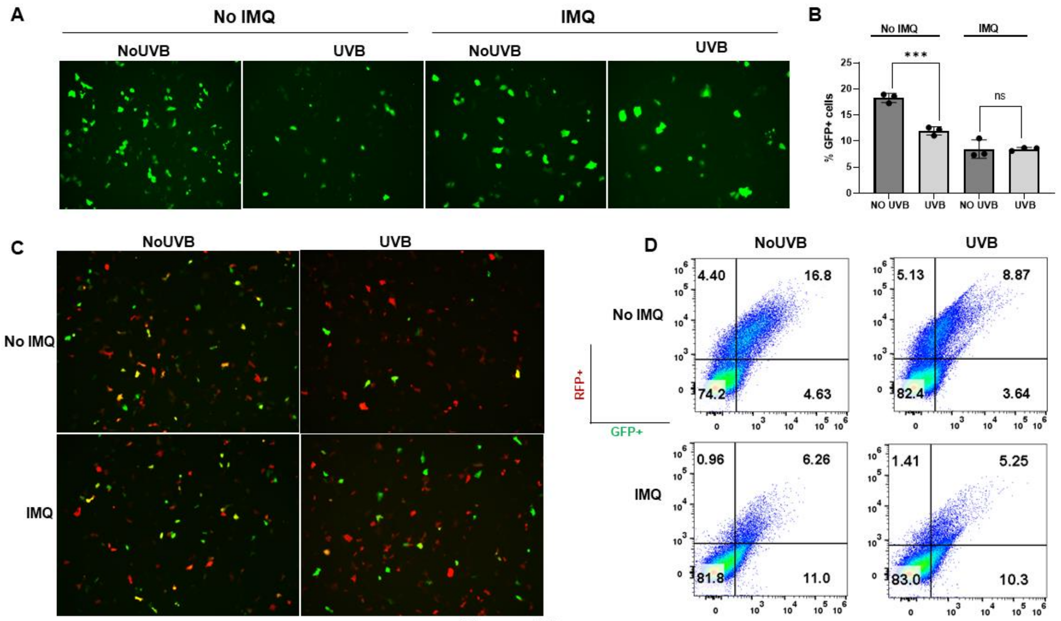

Figure S1

Supplementary Figure S1. Type I IFN agonist imiquimod (IMQ) repairs UVB-induced DNA damage. Transfection of normal and UVB treated plasmid in keratinocytes in the presence or absence of IMQ, showed the ability of repairing UVB treated pEGFP plasmid gene. UVB treated plasmid expression documented by microscopy and FACS analysis compared to control plasmid (Figure S1 A, B). The decreased expression of UVB-treated pEGFP plasmid was fully rescued in the presence of IMQ. These results were confirmed in a separate experiment where pEGFP was co-transfected with untreated DsRedexpress plasmid (Figure S1 C,D).
